# Retinoic acids and nuclear receptor signaling in liver development: Pathogenic roles in liver diseases

**DOI:** 10.1002/pdi3.29

**Published:** 2023-09-27

**Authors:** Wen Jia, Yang Bi

**Affiliations:** ^1^ Stem Cell Biology and Therapy Laboratory Ministry of Education Key Laboratory of Child Development and Disorders Chongqing Key Laboratory of Pediatrics National Clinical Research Center for Child Health and Disorders The Children's Hospital of Chongqing Medical University Chongqing China

**Keywords:** hepatitis, hepatocellular carcinoma, liver, liver fibrosis, retinoic acid

## Abstract

Retinoic acid (RA) serves as a metabolic intermediate of vitamin A. It plays a crucial physiological role in regulating cell proliferation, differentiation, apoptosis, embryonic development, and immunomodulation. Once vitamin A enters the body in the form of retinol, it undergoes conversion into RA through the intestinal epithelium and liver. Subsequently, it interacts with retinoic acid receptors and retinoid X receptors within the cell nucleus, thereby regulating gene expression. Throughout liver development, RA exerts precise temporal control, stimulating liver growth, inducing RALDH2 expression in liver somatic epithelial cells, and influencing hepatocyte differentiation. Recent studies have consistently demonstrated the indispensable connection between RA deficiency and the development of liver diseases, including nonalcoholic fatty liver disease, chronic hepatitis, liver fibrosis, and liver tumors. Studying the mechanisms underlying the relationship between RA and disease can enhance our understanding and improve disease treatment. This paper provides a comprehensive review of the role of RA signaling in liver development and liver diseases.

## 1 INTRODUCTION

**FIGURE 1:**
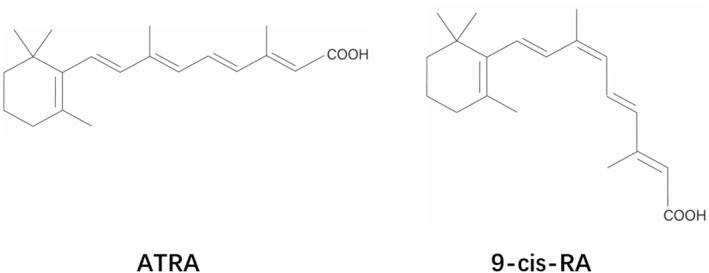
Chemical structures of ATRA and 9‐cis‐RA. ATRA, all‐trans retinoic acid.

Retinoic acid (RA), a metabolic intermediate of vitamin A, is a crucial physiological regulator that controls cell proliferation, differentiation, and apoptosis. It plays a significant role in vertebrate embryonic development, bone growth, hematopoiesis, vision, reproduction, and metabolism.^1^, ^2^ Biologically active endogenous RAs include all‐trans retinoic acid (ATRA), 9‐cis retinoic acid (9CRA), 11‐cis retinaldehyde, and 3,4‐dihydro retinoic acid^3^ (Figure 1). RA is a fast‐dispersing signaling molecule that recognizes and regulates gene expression in cells by binding to specific nuclear receptors. Its ability to regulate cell proliferation and apoptosis suggests that RA could be beneficial for cancer prevention and treatment. Currently, it is extensively utilized to treat various dermatological diseases, acute promyelocytic leukemia, and other conditions. The involvement of RA in liver development, differentiation, and disease regulation is substantial. Therefore, this paper comprehensively reviews the role of RA signaling in liver development and disease.

## 2 RA SIGNALING

### 2.1 Retinol metabolism

Vitamin A exists in various forms outside the body, including carotenoids found in plants like carrots and lettuce, and retinol present in animal liver and tuna.^4^ The metabolism of dietary retinyl esters (RES) and dietary β‐carotene results in the production of the primary parent retinoid in the natural cycle.^5^ Upon entering the body, vitamin A from food sources undergoes initial absorption by intestinal epithelial cells, followed by metabolism to RES. Subsequently, it gets encapsulated into chylomicron particles, which are transported to the lymphatic system or portal vein.^6^, ^7^ Some of the chylomicron particles reach the liver, where they are converted into retinol, and subsequently transferred to lipid‐like cells or Golgi bodies for storage. When the body requires vitamin A, retinol binds with retinol‐binding protein (RBP) in the liver, forming the retinol‐RBP complex. A fraction of this complex travels through the bloodstream, reaching the digestive tract and skin, while the remainder enters the small intestine with bile, facilitating transportation to the cells that need vitamin A.^3^ The retinol‐RBP complex facilitates retinol's entry into the cell by binding to the cell surface. Subsequently, intracellular alcohol dehydrogenase oxidizes retinol, leading to either reversible conversion to retinaldehyde or irreversible conversion to RA. These products then interact with DNA receptors in the nucleus, regulating the expression of pertinent genes.^8^

The liver has a pivotal role in regulating vitamin A metabolism and managing chronic liver diseases, including viral hepatitis, alcoholic liver disease,^9^ non‐alcoholic fatty liver disease (NAFLD), and non‐alcoholic steatohepatitis (NASH).^10^ The liver produces bile, which aids in the intestinal absorption of fat‐soluble nutrients, including vitamin A. Additionally, the liver synthesizes retinol‐binding protein 4 (RBP4), responsible for delivering vitamin A to peripheral tissues in the form of retinol. Moreover, the liver houses the highest concentration of hepatic stellate cells (HSCs), acting as the primary storage site for vitamin A in the form of retinol.^11^

RA is a crucial micronutrient for children's growth and development, playing an indispensable role in cell proliferation, differentiation, and apoptosis. Vitamin A and its derivatives find application in treating various conditions, including bone growth disorders, acne, vision problems, acute promyelocytic leukemia, liver diseases, and tumors.

### 2.2 RARs and signaling pathways

**FIGURE 2:**
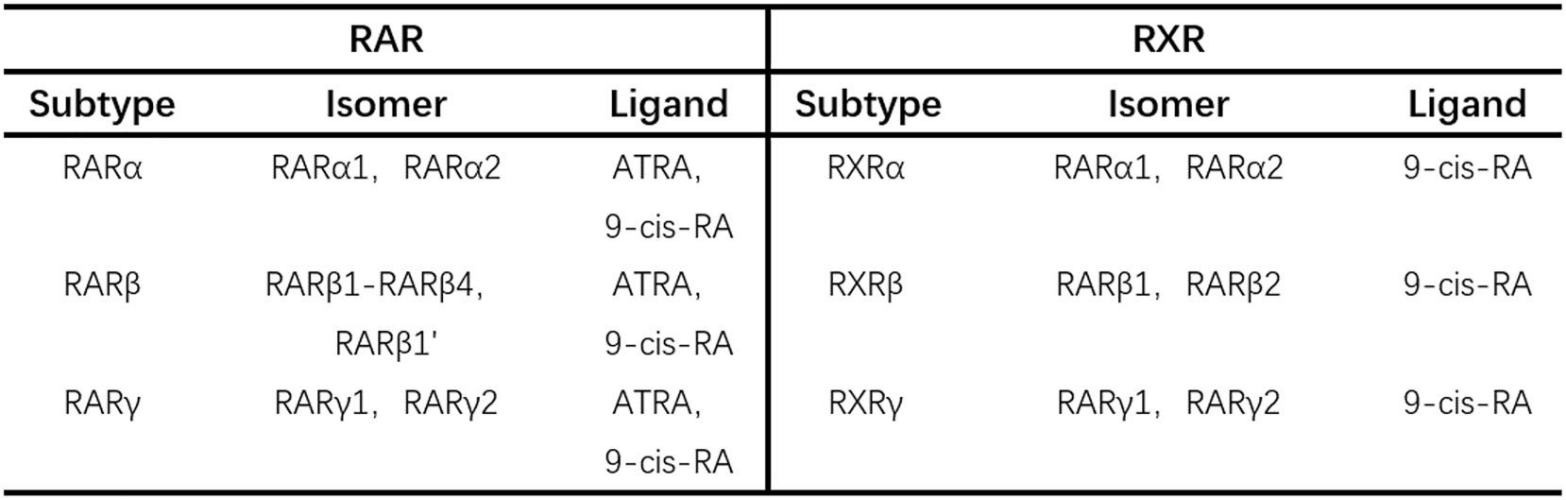
RAR and RXR Subtypes, Isomers, and Their Ligands. RAR, retinoic acid receptor; RXR, retinoid X receptor.

**FIGURE 3:**
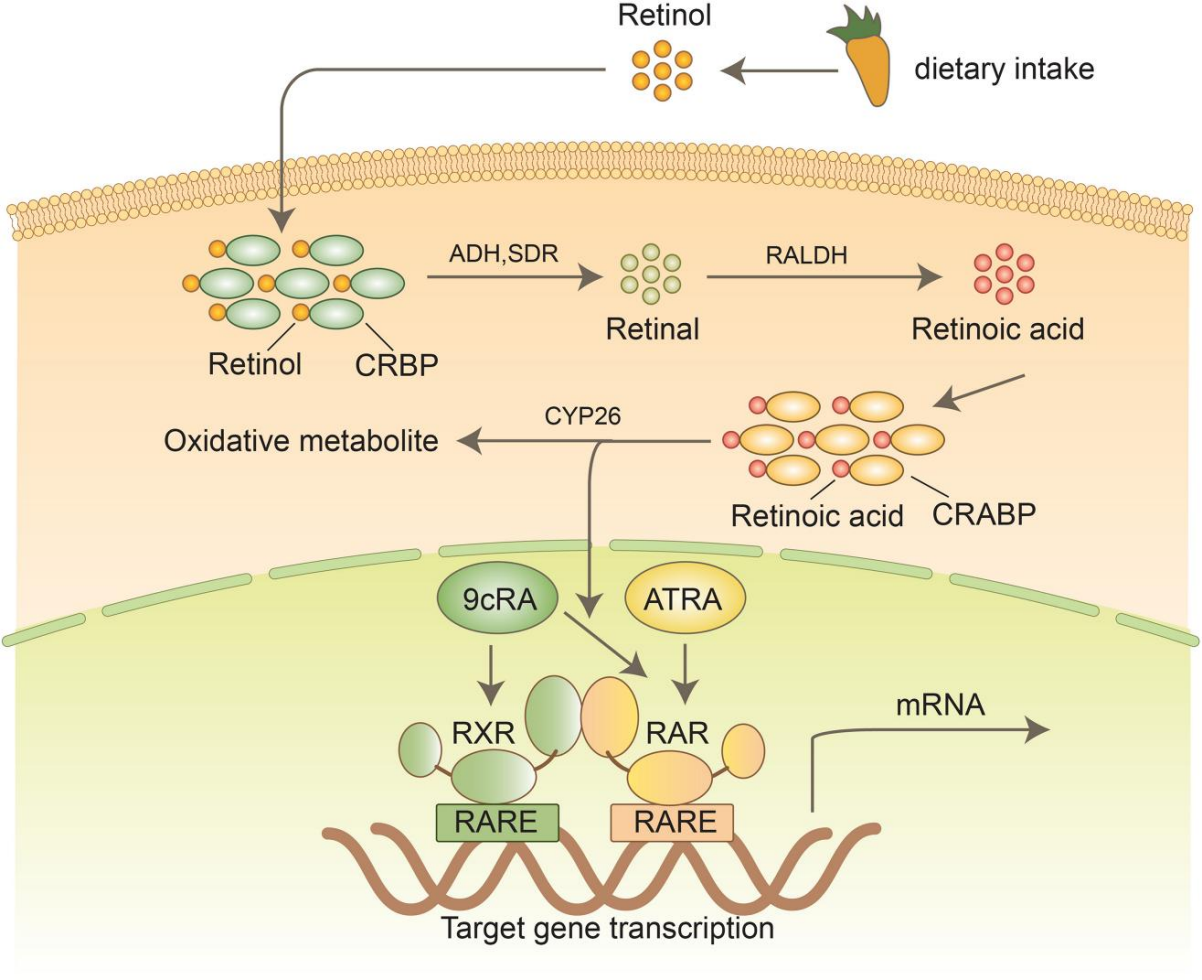
General mechanism of nuclear receptor signaling for retinoic acid.

RA enters the nucleus and directly binds to target genes via nuclear receptors known as retinoic acid receptors (RARs). These receptors belong to two families: RARs and retinoid X receptors (RXRs) (Figure 2). They serve as significant regulators of human gene expression and essential drug targets, acting primarily as heterodimers that activate transcription of target genes in the presence of their ligand, ATRA.^12^ Endogenous RA levels are regulated by RA synthesizing and degrading enzymes. Upon binding to nuclear RAR and DNA response elements in the nucleus, RA activates or represses relevant target genes, thus regulating transcription and enzyme expression.^13^, ^14^ When RXR binds to DNA, it, along with RAR and several other nuclear receptors, forms a heterodimer, facilitating DNA binding to various classes of nuclear receptors. When RA binds to this RAR/RXR heterodimer, it regulates DNA components, ultimately leading to the recruitment of transcriptional co‐activators and the initiation of transcription^15^ (Figure 3).

In this RAR pathway, when ligands are absent or inverse agonists are present, RAR exhibits strong inhibitory activity due to the recruitment and binding of co‐repressors, leading to the formation of co‐repressor complexes in the promoter regions of target genes.^16^ Upon activation by RA and binding to intracellular RA‐binding protein II, RAR undergoes a conformational change, altering the chromosome's repressor structure, dissociating from the repressor, and recruiting coactivator ATP‐dependent remodeling complexes, which displace the inhibitory nucleosome within the proximal promoter region, thus facilitating the insertion of the transcription machinery into the promoter. Subsequently, chromatin unwinds to facilitate transcription.^17^

In vitro, the activation of the RAR‐RXR heterodimer can be accomplished by the individual binding of RAR and RXR ligands to their respective receptors. However, this does not seem possible with RXR‐specific ligands (epinephrine‐like) in cellular environments lacking RAR agonists. Consequently, the only way for RXR to regulate its own ligand activation in RAR‐RXR heterodimers is through a synergistic interaction with RAR ligands.^18^

### 2.3 RA and growth

RA plays a vital role in human embryonic development, particularly in brain, craniofacial, and limb function, and morphogenesis. Additionally, it acts as a crucial component of cell‐to‐cell signaling pathways during human organ development.^19^ To prevent deficiency and toxicity, concentrations of vitamin A must be tightly regulated within a narrow range. Consequently, adding vitamin A or RA to the embryo is likely to induce teratogenic effects, leading to significant organ alterations.^20^

RA plays a pivotal role in early embryonic development by providing instructive signals for posterior neural ectoderm and foregut endoderm, and permissive signals for mesodermal differentiation of the main stem. Additionally, it contributes to the development of organs like the eye during the later stages of embryonic development.^21^ Signaling molecular gradients drive morphogenesis and pattern formation in the embryo. These gradients also regulate specific gene expression programs, ultimately determining cellular fate.^22^ RAR‐RXRs play pivotal roles in embryonic development patterns, tissue differentiation, and organ development through gene regulation. Additionally, they are involved in metabolic regulation, tissue differentiation, regeneration, and repair after birth.^23^ In the fetus, RBP4 is secreted by hepatocytes and visceral yolk sacs, and it transports retinol to target tissues for ATRA synthesis, thereby promoting ATRA production. Adult mice with RBP4 deletion mutations exhibited reduced fertility and experienced severe structural changes in the retina, leading to vision loss.^24^ Blocking caudal Fgf8 expression enables RA to regulate trunk growth adequately, ensuring normal spinal neuronal differentiation and somatogenesis.^25^ Thus, it is evident that retinoids, RA, and its derivatives play a crucial role in embryonic development, with RA being essential for the development of several organs, including the hindbrain, spinal cord, heart, eyes, bones, forelimb buds, lungs, pancreas, and genitourinary tract.^21^

## 3 RA AND LIVER DEVELOPMENT

### 3.1 RA and embryonic liver development

Hepatocytes in the embryonic stage differentiate initially from the endoderm.^26^ Totipotent stem cells of the blastocyst endodermal cell mass differentiate into pluripotent tissue‐specific progenitors. Subsequently, the medial and lateral structural domains fuse during foregut closure, leading to endodermal cells differentiating into liver under the influence of induced signals and genetic regulatory factors.^27^ Fibroblast growth factor and BMP4 signals from the mesoderm trigger the formation of the liver from the foregut endoderm at somatic cell stages 5–6.^28^ Hepatoblasts form liver buds by changing their morphology to columnar and invading the mesenchyme of the transverse septum. In this process, mechanisms coordinate nuclear migration, proliferation, loss of intercellular adhesion, hepatoblast migration, and tissue‐specific differentiation. Studies in mutant mice have revealed that the formation of liver buds is tightly controlled by a network of transcription factors. Initially, Wnt signaling maintains foregut identity in an inhibitory form and enables hepatic induction. Subsequently, it promotes liver bud emergence and differentiation.^29^, ^30^

Ijpenberg et al.^31^ conducted experiments that confirmed the low proliferation of quail liver explants in culture. However, the addition of RA to the culture medium resulted in increased cell proliferation, even in the presence of citral, an RA synthesis inhibitor. Therefore, RA levels in the liver environment may stimulate liver growth and induce RALDH2 expression in liver somatic epithelial cells. RXRα‐deficient embryos have underdeveloped livers, and inhibiting RALDH2 expression at early liver development stages (HH14‐15) results in hypoplastic livers. However, the same treatment at later stages of development (HH18) has no effect, indicating a very precise developmental window during which RA signaling is required.^31^

### 3.2 RA and hepatocyte differentiation

The liver possesses the remarkable ability to regenerate after partial hepatectomy or injury, and liver stem cells play a crucial role in its development.^32^, ^33^ The liver originates from embryonic ventral foregut endodermal cells, which are considered to be multipotent stem cells that differentiate specifically into the liver to form hepatic progenitors. The hepatic progenitor base receives signaling stimuli from the mesenchyme of the transverse septum, leading to further differentiation and development.^34^ The majority of liver‐related stem cell research has been centered around fetal‐derived hepatic stem cells or oval cells. During embryogenesis, the cellular composition of the liver is primarily hepatoblasts, which commence the differentiation into hepatocytes and cholangiocytes on embryonic day 14 (E14).^9^

Liver differentiation has been associated with RA.^35^ Falasea et al.^36^ observed that adult hepatocytes, when cultured in vitro, underwent a transition from their original morphology to a fibroblast‐like morphology and structure, indicating a dedifferentiated state. However, upon the addition of 5 μmol/L RA to the dedifferentiated hepatocytes, the characteristic structure of mature hepatocytes was restored within 48 h, gradually transitioning from the altered morphology to the original structure. This suggests that RA influences hepatocyte differentiation and can promote the cells to regain their original differentiation and maturity. To further investigate the impact of RA on liver stem cells, Huang et al.^15^ established reversibly stable hepatocyte progenitor cells (HPCs) in E14.5 mouse fetal livers. They subsequently treated the cells with ATRA and 9CRA, resulting in a reduction of early progenitor cell markers' expression and a notable increase in mature hepatocyte markers' expression. This suggests that both retinoids, ATRA and 9CRA, induced terminal hepatic differentiation of HPCs derived from mouse fetal livers, indicating that the RA signaling pathway plays an essential role in regulating liver differentiation.

### 3.3 RA and lipid metabolism

Vitamin A (VA) has demonstrated its ability to regulate body weight, glucose, and fat metabolism in animal models.^37^ Treatment with RA decreases the body weight of mice that have gained weight from food consumption and reduces the weight of white adipose tissue in lean mice.^38^, ^39^ RA stimulates lipolysis, activates PPARβ/δ and RARs, and inhibits fat accumulation in mature white adipocytes. In animal models, fasting and refeeding lead to changes in the expression levels of RARs and RXRs in mice, and genetic alteration of the RXRs gene causes changes in lipid and glucose metabolism in mice. RXRs and RARs act as mediators of RA and VA signaling and participate in a broad spectrum of physiological processes, including the regulation of metabolic homeostasis.^40^ Previous studies have demonstrated that both the VA status and the activation of RARs and RXRs by RA and synthetic agonists modify lipid and glucose metabolism, implying a crucial role for RARs and RXRs gene expression in metabolic regulation. The RA signaling system interacts with insulin, and numerous target genes of RARs and RXRs are similarly regulated by the insulin signaling pathway.^41^

Retinoids and retinoid receptors regulate the expression of key genes involved in hepatic lipid metabolism. Earlier research has demonstrated that upregulation of RARβ, which encodes fibroblast growth factor 21, through ATRA, enhances total energy expenditure by promoting hepatic fatty acid (FA) oxidation and ketone body production in male mice.^42^ Trasino et al.^43^ discovered a strong negative correlation between hepatic retinol, retinyl palmitate, and retinoid receptor β2 mRNA levels, and disease severity in patients with NAFLD. Moreover, RA induces “browning” of white adipose tissue, leading to a reduction in the accumulation of excess triglycerides.^44^

## 4 RETINOIDS AND LIVER DISEASE

Recent research has demonstrated a substantial correlation between vitamin A levels and the onset and progression of liver injury. While ATRA successfully prevents the onset of liver fibrosis, serum vitamin A levels have been reported to steadily decline with the severity of chronic liver disease.^45^ Generally speaking, patients with persistent cholestasis have low vitamin A levels. RA efficiently reduces or even prevents hepatic fibrosis, according to several studies employing animal models of chronic cholestasis. It also controls the hepatic immunological response to cholestatic damage.^46^ Patients with nonalcoholic fatty liver disease had reduced blood RA levels, according to clinical trials. Further research revealed a negative correlation between RA levels and hepatic triglyceride levels, the degree of hepatic steatosis, and liver damage.^47^ Due to its cellular regulatory effects, RA finds extensive application in the treatment of various types of cancer, including liver cancer, leukemia, and osteosarcoma.

### 4.1 RA and metabolic diseases of the liver

The liver is a vital organ capable of performing numerous metabolic activities essential for maintaining metabolic homeostasis, such as glycolysis, gluconeogenesis, lipogenesis, and glycogen synthesis. NAFLD represents the hepatic manifestation of the metabolic syndrome, encompassing a spectrum of disorders that range from benign hepatic steatosis to NASH. It is frequently associated with systemic metabolic disturbances and substantial alterations in various metabolic pathways.^11^ These metabolic pathways are intricately interconnected and play a crucial role in either preventing or accelerating the disease's onset. NAFLD is characterized by the accumulation of fats in the liver, particularly non‐esterified FAs, triglycerides, and non‐esterified cholesterol.^48^, ^49^ Excess metabolizing substrates, including FA and carbohydrates, surpassing the liver's processing capacity result in elevated formation of reactive oxygen species (ROS), byproducts, and lipid peroxidation. This, in turn, causes hepatocyte damage, inflammation, and eventual cell death.^50^

Ultrasound examinations revealed a strong association between low serum retinol levels and insulin resistance in 11%–36% of morbidly obese adults with NAFLD. Additionally, hepatic retinol levels displayed a notable negative correlation with the histologic classification of the disease.^51^ NAFLD patients exhibited significantly lower circulating ATRA levels compared to controls. Furthermore, ATRA concentrations and RXR levels displayed a negative correlation with hepatic fat content, hepatic steatosis, and the severity of liver disease. NAFLD patients exhibited significantly higher expression levels of vitamin A storage‐related genes (LRAT and DGAT1), retinaldehyde dehydrogenases 1 and 3 (RALDH1 and 3), and degradation genes (Cyp26A).^52^ The conversion of retinol to retinol and ultimately to RA is accelerated in NASH liver tissue. This sustained state of active RA metabolism leads to the loss of RA in NASH liver tissue, potentially contributing to the progression of steatohepatitis to liver cirrhosis and hepatocellular carcinoma (HCC).^53^

### 4.2 RA and hepatitis

Hepatitis C virus (HCV) is one of the most dangerous and potent hepatophilic viruses causing human infections. HCV causes chronic liver infection, which can result in liver failure, cirrhosis, HCC, and liver inflammation.^54^, ^55^ RA, a ligand for RAR and RXR, significantly inhibits extracellular HCV RNA in a dose‐dependent manner. These substances significantly reduced the amount of HCV RNA and NS5A protein in cells carrying subgenomic HCV replication factor RNA, thus impacting HCV RNA replication.^56^ Additionally, RA seemed to attenuate the post‐viral replication phase, as the reduction in intracellular HCV RNA was less pronounced compared to the reduction in extracellular HCV RNA.

Retinoic acid‐inducible gene‐I (RIG‐I) is a crucial type I interferon‐dependent gene that triggers the antiviral innate immune response. RIG‐I ligand (5′PPP‐d sRNA) treatment significantly increased the levels of interferon and interferon in LX‐2 cells compared to control cells.^57^ Toll‐like receptor 3 and interferon regulatory factor‐7 are critical regulators of the interferon signaling pathway, and their activation in LX‐2 cells results from RIG‐I activation. Additionally, gastrointestinal glutathione peroxidase (GI‐GPX) represents a potential target for treating HCV infection.^58^ There is a negative correlation between intracellular HCV RNA and GI‐GPX gene and protein expression in various HCV replicating cell lines.^59^ As the GI‐GPX promoter contains a RA response element, ATRA promotes high expression of GI‐GPX in replicating cells, leading to successful downregulation of HCV production.^60^

### 4.3 RA and liver fibrosis

The primary contributing factor to liver fibrosis is the excessive accumulation of extracellular matrix (ECM) proteins. In liver fibrosis, ECM accumulates in the liver, disrupting the natural liver structure due to continuous injury and inflammation, resulting in hepatocellular dysfunction and liver cirrhosis.^61^ HSCs are the main cell type responsible for ECM production in the liver, and they also play a crucial role in the formation and development of liver fibrosis.^62^

The transforming growth factor (TGF)‐β1‐Smad signaling pathway is a classical mechanism that promotes liver fibrosis. This pathway acts as a potent fibrogenic cytokine. Upon activation, the TGF binds to the receptor and transmits relevant signals.^63^ ATRA inhibits the transcription of downstream genes TGF‐1 and activator protein‐1, promotes mRNA expression of fibrinolytic genes, and blocks HSC proliferation and collagen synthesis.^64^ Matrix metalloproteinases (MMPs) are mainly responsible for degrading matrix proteins, particularly collagen and non‐collagenous proteins. ATRA inhibits TGF‐β1, which triggers the production of MMPs that break down collagen, thereby reducing the expression of downstream fibrogenic genes involved in liver fibrosi.^65^ Through the TGF‐Smad signaling pathway, ATRA controls the ratio of Th17/Treg cells, induces Treg cell differentiation, and blocks thioredoxin interacting protein (TXNIP) to inhibit HSC activation. This inhibits collagen production, slows down hepatic fibrosis development, and protects the liver.^66^ RA effectively inhibits the expression of various collagens, including collagen type III, collagen type 1A1, and collagen type III. Additionally, it inhibits the expression of various biomolecules, such as α‐SMA, tumor necrosis factor‐α, and IL‐6, which may play a protective role in fibrotic diseases like hepatic fibrosis and renal fibrosis.^67^

Liver fibrosis is primarily induced by HSC activation, and the key approach to halt disease progression involves mitigating oxidative stress in the liver. ATRA prevents the onset and progression of hepatic fibrosis by reducing oxidative stress and inhibiting HSC activation through downregulation of TXNIP protein expression.^68^ ATRA inhibits oxidative stress, downregulates ROS, and thereby prevents HSC activation.

The Wnt signaling pathway plays a vital role in intracellular signaling, primarily regulating early embryonic cell development. Dysregulation or aberrant activation of this pathway can contribute to fibrosis and the development of various diseases.^69^ The Wnt/β‐catenin pathway is a prominent signaling pathway currently under investigation.^70^ Upon initiation of this signaling pathway, β‐catenin stability in the cytoplasm increases and accumulates, leading to its translocation to the nucleus, where it triggers the transcription of relevant target genes. Several target genes closely associated with liver fibrosis include type I collagen, cell cycle proteins, urokinase‐type plasminogen activator receptor, and the MMP family.^71^

Retinoic acid’s receptor‐related orphan receptors (ROR) exhibits widespread distribution in various body tissues, influencing essential processes like inflammatory responses, lipid metabolism, and neural tissue formation.^72^, ^73^ The imbalance in inflammatory response and lipid metabolism control plays a crucial role in determining the rate of liver fibrosis development. ROR regulates the expression of target genes involved in lipid metabolism, thereby promoting lipid metabolism stabilization, making it a potential key factor in preventing liver fibrosis.

### 4.4 RA and liver tumors

HCC is a prevalent malignancy worldwide, often linked to chronic inflammation and hepatic cirrhosis resulting from recurrent hepatitis B or C virus infections.^74^ Due to the critical function of RA and its receptor in normal cell proliferation, differentiation, and death, the aberrant expression and function of these molecules are strongly associated with the development of several human malignancies, including HCC.^75^ RA deprivation has an effect on the development of HCC in both chronic liver disease humans and animals exposed to carcinogens. Moreover, exogenous RA therapy has demonstrated the ability to decelerate HCC development in humans and animals alike.^76^

HCC can also be inadvertently stimulated by the activation of HSCs. RA deprivation has an effect on the development of HCC in both chronic liver disease humans and animals exposed to carcinogens.^53^ Yanagitani et al.^77^ discovered through experimentation that HCC was more prevalent in transgenic mice with hepatocyte‐specific RA signaling inhibition. Epidemiological statistics show that persons with lower blood retinol concentrations who are positive for the hepatitis B surface antigen have a sevenfold increased chance of developing hepatocellular cancer. Moreover, retinol prevents carcinogenesis in several organs, including the stomach, breasts, lungs, prostate, and liver. The activation of HSCs and the progression of liver disease are associated with the loss of fat droplets containing vitamin A. This highlights that reduced retinol levels are a significant risk factor for HCC.^78^

Autophagy is a highly conserved cellular self‐digestive process that degrades modified, excess, or damaged cellular macromolecules and entire organelles. It plays a crucial role in cell development, differentiation, survival, and maintaining cellular homeostasis in vivo.^79^ Fang et al.^80^ discovered that ATRA enhances autophagy levels in Hepa1‐6 and HepG2 cells. Moreover, downregulating autophagy may reverse the behavior of ATRA‐treated HCC cells by mediating epithelial‐mesenchymal transition. These results indicate that ATRA inhibits the proliferation, migration, and invasion of HCC cells while promoting apoptosis and differentiation.

Ferroptosis is a form of programmed cell death triggered by excessive iron accumulation. Sun et al.^81^ detected differentially expressed genes between HCC and normal liver tissues, revealing 53 genes associated with ferroptosis. Treating HCC cells with ATRA downregulated the majority of genes related to ferroptosis. Moreover, a specific inhibitor targeting ferritin restored the ATRA‐regulated expression of ferroptosis‐related genes, leading to a decrease in levels of lipid ROS and MDA. Consequently, this inhibition effectively counteracted ATRA‐induced ferroptosis in HCC cells. These findings imply that ATRA could influence the malignant biological characteristics of HCC by modulating the ferroptosis pathway.

## 5 SUMMARY

Liver diseases, particularly hepatitis, cirrhosis, and liver cancer, have become increasingly prevalent in today's society, posing significant threats to people's lives and health. Research on liver development and the treatment of liver diseases in children has gained significant attention. The above introduction reveals that RA signaling plays a role in liver development, differentiation, and disease progression. It affects various aspects, including cell growth, apoptosis, lipid metabolism, signaling pathways, autophagy, etc. These findings offer valuable insights for studying potential liver disease treatments. However, the complexity of the various regulatory mechanisms involved in RA in vivo requires further in‐depth studies before clinical applications can be considered.

## AUTHOR CONTRIBUTIONS

**Wen Jia**: Writing ‐ original draft. **Yang Bi**: Writing ‐ review & editing.

## CONFLICT OF INTEREST STATEMENT

Yang Bi is a member of Pediatric Discovery Editorial Board. To minimize bias, she was excluded from all editorial decision‐making related to the acceptance of this article for publication. The other author declares no conflict of interest.

## ETHICS STATEMENT

Not applicable.

## Data Availability

Data sharing is not applicable to this article as no new data were created or analyzed in this study.

## References

[1] Shiota G , Tsuchiya H , Hoshikawa Y . The liver as a target organ of retinoids. Hepatol Res. 2006;36(4):248‐254. 16996300 10.1016/j.hepres.2006.08.010

[2] Sun S.‐Y , Lotan R . Retinoids and their receptors in cancer development and chemoprevention. Crit Rev Oncol Hematol. 2002;41(1):41‐55. 11796231 10.1016/s1040-8428(01)00144-5

[3] Theodosiou M , Laudet V , Schubert M . From carrot to clinic: an overview of the retinoic acid signaling pathway. Cell Mol Life Sci. 2010;67(9):1423‐1445. 20140749 10.1007/s00018-010-0268-z PMC11115864

[4] Carazo A , Macakova K , Matousova K , Krcmova LK , Protti M , Mladenka P . Vitamin A update: forms, sources, kinetics, detection, function, deficiency, therapeutic use and toxicity. Nutrients. 2021;13(5):1703. 34069881 10.3390/nu13051703 PMC8157347

[5] Steinhoff JS , Lass A , Schupp M . Retinoid homeostasis and beyond: how retinol binding protein 4 contributes to health and disease. Nutrients. 2022;15(6):14. 35334893 10.3390/nu14061236 PMC8951293

[6] Gudas LJ . Retinoid metabolism: new insights. J Mol Endocrinol. 2022;69(4):T37‐T49. 35900851 10.1530/JME-22-0082 PMC9561048

[7] Reboul E . Proteins involved in fat‐soluble vitamin and carotenoid transport across the intestinal cells: new insights from the past decade. Prog Lipid Res. 2023;89:101208. 36493998 10.1016/j.plipres.2022.101208

[8] Andre A , Ruivo R , Fonseca E , Froufe E , Castro LFC , Santos MM . The retinoic acid receptor (RAR) in molluscs: function, evolution and endocrine disruption insights. Aquat Toxicol. 2019;208:80‐89. 30639747 10.1016/j.aquatox.2019.01.002

[9] Hyun J , Han J , Lee C , Yoon M , Jung Y . Pathophysiological aspects of alcohol metabolism in the liver. Int J Mol Sci. 2021;22(11):5717. 34071962 10.3390/ijms22115717 PMC8197869

[10] Cotter TG , Rinella M . Nonalcoholic fatty liver disease 2020: the state of the disease. Gastroenterology. 2020;158(7):1851‐1864. 32061595 10.1053/j.gastro.2020.01.052

[11] Saeed A , Dullaart RPF , Schreuder T , Blokzijl H , Faber KN . Disturbed vitamin A metabolism in non‐alcoholic fatty liver disease (NAFLD). Nutrients. 2017;10(1):29. 29286303 10.3390/nu10010029 PMC5793257

[12] Vucetic Z , Zhang Z , Zhao J , Wang F , Soprano KJ , Soprano DR . Acinus‐S' represses retinoic acid receptor (RAR)‐regulated gene expression through interaction with the B domains of RARs. Mol Cell Biol. 2008;28(8):2549‐2558. 18250153 10.1128/MCB.01199-07 PMC2293115

[13] Sessler RJ , Noy N . A ligand‐activated nuclear localization signal in cellular retinoic acid binding protein‐II. Mol Cell. 2005;18(3):343‐353. 15866176 10.1016/j.molcel.2005.03.026

[14] O'Connor C , Varshosaz P , Moise AR . Mechanisms of feedback regulation of vitamin A metabolism. Nutrients. 2022;21(6):14. 10.3390/nu14061312 PMC8950952 35334970

[15] Huang J , Bi Y , Zhu GH , et al. Retinoic acid signalling induces the differentiation of mouse fetal liver‐derived hepatic progenitor cells. Liver Int. 2009;29(10):1569‐1581. 19737349 10.1111/j.1478-3231.2009.02111.x

[16] Clarke N , Germain P , Altucci L , Gronemeyer H . Retinoids: potential in cancer prevention and therapy. Expet Rev Mol Med. 2004;6(25):1‐23. 10.1017/S1462399404008488 15569396

[17] Magomedova L , Tiefenbach J , Zilberman E , et al. ARGLU1 is a transcriptional coactivator and splicing regulator important for stress hormone signaling and development. Nucleic Acids Res. 2019;47(6):2856‐2870. 30698747 10.1093/nar/gkz010 PMC6451108

[18] Gutierrez‐Mazariegos J , Schubert M , Laudet V . Evolution of retinoic acid receptors and retinoic acid signaling. Subcell Biochem. 2014;70:55‐73. 24962881 10.1007/978-94-017-9050-5_4

[19] Sandell LL , Sanderson BW , Moiseyev G , et al. RDH10 is essential for synthesis of embryonic retinoic acid and is required for limb, craniofacial, and organ development. Genes Dev. 2007;21(9):1113‐1124. 17473173 10.1101/gad.1533407 PMC1855236

[20] Bowles J , Knight D , Smith C , et al. Retinoid signaling determines germ cell fate in mice. Science. 2006;312(5773):596‐600. 16574820 10.1126/science.1125691

[21] Duester G . Retinoic acid synthesis and signaling during early organogenesis. Cell. 2008;134(6):921‐931. 18805086 10.1016/j.cell.2008.09.002 PMC2632951

[22] Sosnik J , Zheng L , Rackauckas CV , et al. Noise modulation in retinoic acid signaling sharpens segmental boundaries of gene expression in the embryonic zebrafish hindbrain. Elife. 2016;5:e14034. 27067377 10.7554/eLife.14034 PMC4829421

[23] Ghyselinck NB , Duester G . Retinoic acid signaling pathways. Development. 2019;146(13). 10.1242/dev.167502 PMC6633611 31273085

[24] Shannon SR , Moise AR , Trainor PA . New insights and changing paradigms in the regulation of vitamin A metabolism in development. Wiley Interdiscip Rev Dev Biol. 2017;6(3). 10.1002/wdev.264 PMC5911347 28207193

[25] Brondani V , Klimkait T , Egly JM , Hamy F . Promoter of FGF8 reveals a unique regulation by unliganded RARalpha. J Mol Biol. 2002;319(3):715‐728. 12054865 10.1016/S0022-2836(02)00376-5

[26] Nowotschin S , Hadjantonakis AK . Guts and gastrulation: emergence and convergence of endoderm in the mouse embryo. Curr Top Dev Biol. 2020;136:429‐454. 31959298 10.1016/bs.ctdb.2019.11.012

[27] Han L , Chaturvedi P , Kishimoto K , et al. Single cell transcriptomics identifies a signaling network coordinating endoderm and mesoderm diversification during foregut organogenesis. Nat Commun. 2020;11(1):4158. 32855417 10.1038/s41467-020-17968-x PMC7453027

[28] Kopp JL , Grompe M , Sander M . Stem cells versus plasticity in liver and pancreas regeneration. Nat Cell Biol. 2016;18(3):238‐245. 26911907 10.1038/ncb3309

[29] McLin VA , Rankin SA , Zorn AM . Repression of Wnt/beta‐catenin signaling in the anterior endoderm is essential for liver and pancreas development. Development. 2007;134(12):2207‐2217. 17507400 10.1242/dev.001230

[30] Monga SP , Monga HK , Tan X , Mule K , Pediaditakis P , Michalopoulos GK . Beta‐catenin antisense studies in embryonic liver cultures: role in proliferation, apoptosis, and lineage specification. Gastroenterology. 2003;124(1):202‐216. 12512043 10.1053/gast.2003.50000

[31] Ijpenberg A , Perez‐Pomares JM , Guadix JA , et al. Wt1 and retinoic acid signaling are essential for stellate cell development and liver morphogenesis. Dev Biol. 2007;312(1):157‐170. 18028902 10.1016/j.ydbio.2007.09.014

[32] Kung JW , Currie IS , Forbes SJ , Ross JA . Liver development, regeneration, and carcinogenesis. J Biomed Biotechnol. 2010;2010:984248. 20169172 10.1155/2010/984248 PMC2821627

[33] Zhu X , Wang W , Zhang X , et al. All‐trans retinoic acid‐induced deficiency of the Wnt/beta‐catenin pathway enhances hepatic carcinoma stem cell differentiation. PLoS One. 2015;10(11):e0143255. 26571119 10.1371/journal.pone.0143255 PMC4646487

[34] Duncan SA . Mechanisms controlling early development of the liver. Mech Dev. 2003;120(1):19‐33. 12490293 10.1016/s0925-4773(02)00328-3

[35] Liu HX , Ly I , Hu Y , Wan YJ . Retinoic acid regulates cell cycle genes and accelerates normal mouse liver regeneration. Biochem Pharmacol. 2014;91(2):256‐265. 25087568 10.1016/j.bcp.2014.07.003 PMC4236911

[36] Falasca L , Favale A , Serafino A , Ara C , Conti Devirgiliis L . The effect of retinoic acid on the re‐establishment of differentiated hepatocyte phenotype in primary culture. Cell Tissue Res. 1998;293(2):337‐347. 9662656 10.1007/s004410051125

[37] Chen W , Chen G . The roles of vitamin A in the regulation of carbohydrate, lipid, and protein metabolism. J Clin Med. 2014;3(2):453‐479. 26237385 10.3390/jcm3020453 PMC4449691

[38] Xue S , Lee D , Berry DC . Thermogenic adipose tissue in energy regulation and metabolic health. Front Endocrinol. 2023;14:1150059. 10.3389/fendo.2023.1150059 PMC10067564 37020585

[39] Malibary MA . Vitamin A: a key inhibitor of adipocyte differentiation. PPAR Res. 2023;2023:7405954. 36776154 10.1155/2023/7405954 PMC9908342

[40] Park S , Lee YJ , Ko EH , Kim JW . Regulation of retinoid X receptor gamma expression by fed state in mouse liver. Biochem Biophys Res Commun. 2015;458(1):134‐139. 25637539 10.1016/j.bbrc.2015.01.082

[41] Chen G . The interactions of insulin and vitamin A signaling systems for the regulation of hepatic glucose and lipid metabolism. Cells. 2021;10(8):2160. 34440929 10.3390/cells10082160 PMC8393264

[42] Blaner WS . Vitamin A signaling and homeostasis in obesity, diabetes, and metabolic disorders. Pharmacol Ther. 2019;197:153‐178. 30703416 10.1016/j.pharmthera.2019.01.006 PMC6520171

[43] Trasino SE , Tang XH , Jessurun J , Gudas LJ . Obesity leads to tissue, but not serum vitamin A deficiency. Sci Rep. 2015;5(1):15893. 26522079 10.1038/srep15893 PMC4629132

[44] Villarroya F , Gavalda‐Navarro A , Peyrou M , Villarroya J , Giralt M . The lives and times of brown adipokines. Trends Endocrinol Metabol. 2017;28(12):855‐867. 10.1016/j.tem.2017.10.005 29113711

[45] Chaves GV , Peres WA , Goncalves JC , Ramalho A . Vitamin A and retinol‐binding protein deficiency among chronic liver disease patients. Nutrition. 2015;31(5):664‐668. 25837210 10.1016/j.nut.2014.10.016

[46] Freund C , Gotthardt DN . Vitamin A deficiency in chronic cholestatic liver disease: is vitamin A therapy beneficial? Liver Int. 2017;37(12):1752‐1758. 28371374 10.1111/liv.13433

[47] Liu Y , Chen H , Wang J , Zhou W , Sun R , Xia M . Association of serum retinoic acid with hepatic steatosis and liver injury in nonalcoholic fatty liver disease. Am J Clin Nutr. 2015;102(1):130‐137. 25948673 10.3945/ajcn.114.105155

[48] Villaca Chaves G , Pereira SE , Saboya CJ , Ramalho A . Non‐alcoholic fatty liver disease and its relationship with the nutritional status of vitamin A in individuals with class III obesity. Obes Surg. 2008;18(4):378‐385. 18264740 10.1007/s11695-007-9361-2

[49] Beydoun MA , Canas JA , Beydoun HA , Chen X , Shroff MR , Zonderman AB . Serum antioxidant concentrations and metabolic syndrome are associated among U.S. adolescents in recent national surveys. J Nutr. 2012;142(9):1693‐1704. 22810988 10.3945/jn.112.160416 PMC3417831

[50] Lu Q , Tian X , Wu H , et al. Metabolic changes of hepatocytes in NAFLD. Front Physiol. 2021;12:710420. 34526911 10.3389/fphys.2021.710420 PMC8437340

[51] Chaves GV , Pereira SE , Saboya CJ , Spitz D , Rodrigues CS , Ramalho A . Association between liver vitamin A reserves and severity of nonalcoholic fatty liver disease in the class III obese following bariatric surgery. Obes Surg. 2014;24(2):219‐224. 24101088 10.1007/s11695-013-1087-8

[52] Clemente C , Elba S , Buongiorno G , Berloco P , Guerra V , Di Leo A . Serum retinol and risk of hepatocellular carcinoma in patients with child‐Pugh class A cirrhosis. Cancer Lett. 2002;178(2):123‐129. 11867196 10.1016/s0304-3835(01)00843-6

[53] Shiota G , Kanki K . Retinoids and their target genes in liver functions and diseases. J Gastroenterol Hepatol. 2013;28(suppl 1):33‐37. 10.1111/jgh.12031 23855293

[54] Chigbu DI , Loonawat R , Sehgal M , Patel D , Jain P . Hepatitis C virus infection: host(‐)virus interaction and mechanisms of viral persistence. Cells. 2019;8(4):376. 31027278 10.3390/cells8040376 PMC6523734

[55] D'Souza S , Lau KC , Coffin CS , Patel TR . Molecular mechanisms of viral hepatitis induced hepatocellular carcinoma. World J Gastroenterol. 2020;26(38):5759‐5783. 33132633 10.3748/wjg.v26.i38.5759 PMC7579760

[56] Murakami Y , Fukasawa M , Kaneko Y , Suzuki T , Wakita T , Fukazawa H . Retinoids and rexinoids inhibit hepatitis C virus independently of retinoid receptor signaling. Microbes Infect. 2014;16(2):114‐122. 24177211 10.1016/j.micinf.2013.10.016

[57] Wang Y , Ye L , Wang X , Li J , Song L , Ho W . Retinoic acid inducible gene‐I (RIG‐I) signaling of hepatic stellate cells inhibits hepatitis C virus replication in hepatocytes. Innate Immun. 2013;19(2):193‐202. 23060457 10.1177/1753425912460414 PMC3935722

[58] Morbitzer M , Herget T . Expression of gastrointestinal glutathione peroxidase is inversely correlated to the presence of hepatitis C virus subgenomic RNA in human liver cells. J Biol Chem. 2005;280(10):8831‐8841. 15623509 10.1074/jbc.M413730200

[59] Bocher WO , Wallasch C , Hohler T , Galle PR . All‐trans retinoic acid for treatment of chronic hepatitis C. Liver Int. 2008;28(3):347‐354. 18290777 10.1111/j.1478-3231.2007.01666.x

[60] Brigelius‐Flohe R , Flohe L . Regulatory phenomena in the glutathione peroxidase superfamily. Antioxidants Redox Signal. 2020;33(7):498‐516. 10.1089/ars.2019.7905 31822117

[61] Seki E , Brenner DA . Recent advancement of molecular mechanisms of liver fibrosis. J Hepatobiliary Pancreat Sci. 2015;22(7):512‐518. 25869468 10.1002/jhbp.245 PMC4668270

[62] Lee YA , Wallace MC , Friedman SL . Pathobiology of liver fibrosis: a translational success story. Gut. 2015;64(5):830‐841. 25681399 10.1136/gutjnl-2014-306842 PMC4477794

[63] Budi EH , Duan D , Derynck R . Transforming growth factor‐beta receptors and Smads: regulatory complexity and functional versatility. Trends Cell Biol. 2017;27(9):658‐672. 28552280 10.1016/j.tcb.2017.04.005

[64] Bejjani F , Evanno E , Zibara K , Piechaczyk M , Jariel‐Encontre I . The AP‐1 transcriptional complex: local switch or remote command? Biochim Biophys Acta Rev Cancer. 2019;1872(1):11‐23. 31034924 10.1016/j.bbcan.2019.04.003

[65] Robert S , Gicquel T , Victoni T , et al. Involvement of matrix metalloproteinases (MMPs) and inflammasome pathway in molecular mechanisms of fibrosis. Biosci Rep. 2016;36(4). 10.1042/BSR20160107 PMC4945993 27247426

[66] Kanki K , Akechi Y , Ueda C , et al. Biological and clinical implications of retinoic acid‐responsive genes in human hepatocellular carcinoma cells. J Hepatol. 2013;59(5):1037‐1044. 23831118 10.1016/j.jhep.2013.06.024

[67] Zhou TB , Drummen GP , Qin YH . The controversial role of retinoic acid in fibrotic diseases: analysis of involved signaling pathways. Int J Mol Sci. 2012;14(1):226‐243. 23344030 10.3390/ijms14010226 PMC3565260

[68] Urvalek AM , Gudas LJ . Retinoic acid and histone deacetylases regulate epigenetic changes in embryonic stem cells. J Biol Chem. 2014;289(28):19519‐19530. 24821725 10.1074/jbc.M114.556555 PMC4094062

[69] Trivedi P , Wang S , Friedman SL . The power of plasticity‐metabolic regulation of hepatic stellate cells. Cell Metab. 2021;33(2):242‐257. 33232666 10.1016/j.cmet.2020.10.026 PMC7858232

[70] Lam AP , Gottardi CJ . Beta‐catenin signaling: a novel mediator of fibrosis and potential therapeutic target. Curr Opin Rheumatol. 2011;23(6):562‐567. 21885974 10.1097/BOR.0b013e32834b3309 PMC3280691

[71] Tao LL , Cheng YY , Ding D , et al. C/EBP‐alpha ameliorates CCl(4)‐induced liver fibrosis in mice through promoting apoptosis of hepatic stellate cells with little apoptotic effect on hepatocytes in vitro and in vivo. Apoptosis. 2012;17(5):492‐502. 22307857 10.1007/s10495-012-0700-y

[72] Gu H , Hu P , Zhao Y , et al. Nuclear receptor RORalpha/gamma: exciting modulators in metabolic syndrome and related disorders. Front Nutr. 2022;9:925267. 35799591 10.3389/fnut.2022.925267 PMC9253614

[73] Kang HS , Okamoto K , Takeda Y , et al. Transcriptional profiling reveals a role for RORalpha in regulating gene expression in obesity‐associated inflammation and hepatic steatosis. Physiol Genom. 2011;43(13):818‐828. 10.1152/physiolgenomics.00206.2010 PMC3132837 21540300

[74] Dhamija E , Paul SB , Kedia S . Non‐alcoholic fatty liver disease associated with hepatocellular carcinoma: an increasing concern. Indian J Med Res. 2019;149(1):9‐17. 31115369 10.4103/ijmr.IJMR_1456_17 PMC6507546

[75] Shimizu M , Sakai H , Moriwaki H . Chemoprevention of hepatocellular carcinoma by acyclic retinoid. Front Biosci. 2011;16(2):759‐769. 10.2741/3718 21196201

[76] Haaker MW , Vaandrager AB , Helms JB . Retinoids in health and disease: a role for hepatic stellate cells in affecting retinoid levels. Biochim Biophys Acta Mol Cell Biol Lipids. 2020;1865(6):158674. 32105672 10.1016/j.bbalip.2020.158674

[77] Yanagitani A , Yamada S , Yasui S , et al. Retinoic acid receptor alpha dominant negative form causes steatohepatitis and liver tumors in transgenic mice. Hepatology. 2004;40(2):366‐375. 15368441 10.1002/hep.20335

[78] Weber D , Grune T . The contribution of beta‐carotene to vitamin A supply of humans. Mol Nutr Food Res. 2012;56(2):251‐258. 21957049 10.1002/mnfr.201100230

[79] Galluzzi L , Green DR . Autophagy‐independent functions of the autophagy machinery. Cell. 2019;177(7):1682‐1699. 31199916 10.1016/j.cell.2019.05.026 PMC7173070

[80] Fang S , Hu C , Xu L , et al. All‐trans‐retinoic acid inhibits the malignant behaviors of hepatocarcinoma cells by regulating autophagy. Am J Transl Res. 2020;12(10):6793‐6810. 33194073 PMC7653590

[81] Sun Y , He Y , Tong J , et al. All‐trans retinoic acid inhibits the malignant behaviors of hepatocarcinoma cells by regulating ferroptosis. Genes Dis. 2022;9(6):1742‐1756. 36157492 10.1016/j.gendis.2022.04.011 PMC9485287

